# Contactless deformation of fluid interfaces by acoustic radiation pressure

**DOI:** 10.1038/s41598-023-39464-0

**Published:** 2023-09-07

**Authors:** Félix Sisombat, Thibaut Devaux, Lionel Haumesser, Samuel Callé

**Affiliations:** GREMAN UMR 7347, Université de Tours, INSA CVL, CNRS, 41000 Blois, France

**Keywords:** Acoustics, Physics, Techniques and instrumentation

## Abstract

Reversible and programmable shaping of surfaces promises wide-ranging applications in tunable optics and acoustic metasurfaces. Based on acoustic radiation pressure, contactless and real-time deformation of fluid interface can be achieved. This paper presents an experimental and numerical study to characterize the spatiotemporal properties of the deformation induced by acoustic radiation pressure. Using localized ultrasonic excitation, we report the possibility of on-demand tailoring of the induced protrusion at water–air interface in space and time, depending on the shape of the input pressure field. The experimental method used to measure the deformation of the water surface in space and time shows close agreement with simulations. We demonstrate that acoustic radiation pressure allows shaping protrusion at fluid interfaces, which could be changed into a various set of spatiotemporal distributions, considering simple parameters of the ultrasonic excitation. This paves the way for novel approach to design programmable space and time-dependent gratings at fluid interfaces.

## Introduction

Contactless deformation of surfaces is a topic of growing interest in the field of tunable optic and acoustic metasurfaces^1–7^, as it allows non-contact modification of the physical geometry of surfaces. In the aim of designing a reversible and programmable patterning process, several strategies have been explored using an external energy such as mechanical static energy^8^, thermal expansion^9^, microfluidics^6,10,11^, chemical^12,13^ or electrical tuning^14^. Among them, the use of fluids reveals to be particularly suitable to achieve dynamically tunable devices^1,7,11,15–19^. Recent advances carried out by Melde et al., showed the possibility of assembling particles at the water surface into a variety of 2D shapes using acoustic holograms and radiation pressure^20^. However, most of these concepts suffer from the use of discrete lattice structure to approximate smooth surfaces, or are constrained by mechanical actuators, resulting in a non-real-time of surface shaping and limiting the range of applications in a time-varying environment.

Among the extensive work on non-contact deformation of soft interfaces^21–25^, a promising approach is to remotely control the shape of plane fluid interfaces into 3D out of plane deformation, using acoustic radiation pressure (ARP). ARP has attracted numerous research in past years^26–29^ due to the variety of applications in biomedical^30–33^, for non-contact surface tension measurement^34–36^, or to realize functions including acoustic tweezer^36,37^, enhanced electrospinning^38^ and acoustic diode^16^. Recent study has highlighted the possibility of controlling the height of the interface deformation induced by the ARP as a function of the transient excitation parameters^39^. Nevertheless, determining the spatiotemporal displacement of an interface exposed to ARP remains challenging due to strong hypothesis set by the analytical model. Numerical works have been carried out to tackle these limitations and have conducted to better understanding of the dynamic of the protrusion induced by ARP^40^. However, to our knowledge, no studies have been carried out on the characteristics of the 3D surface deformation in space and time.

The present study investigates both spatial and temporal characteristics of the surface deformation induced by a transient ARP, as a function of the spatiotemporal characteristics of the incident pressure field. It provides new insights for novel application of the ARP for on-demand deformation of fluid interfaces.

Based on theoretical formulation of the ARP induced by a transient ultrasonic excitation, we model the induced surface displacement using Finite Element Method (FEM). Measurements using a polychromatic confocal displacement sensor are performed to measure the time evolution of the interface displacement induced by the transient ARP. The global shape of the deformation is investigated performing a scan over the surface. Several spatiotemporal distributions of the incident pressure field are investigated.

## Results

### Langevin radiation pressure

In a homogeneous medium, considering an acoustic radiation pressure exerted on an interface, as well as an isentropic change of state, the Lagrangian form of the equation of motion is given by^41^:

1 $$\frac{\partial }{\partial t}\left( {\nabla_{L} {\mathbf{x}}_{L} \cdot \frac{{\partial {\mathbf{x}}_{L} }}{\partial t}} \right) = - \nabla_{L} \left( {\int\limits_{{p_{i0} }}^{{p_{L} }} {\frac{dp}{\rho }} - \frac{1}{2}v_{L}^{2} - {\text{g}} \cdot {\mathbf{x}}_{L} \left( {{\mathbf{a}},t} \right)} \right),$$

where ${\mathbf{a}}$ denotes the Lagrangian coordinates, $p_{i0}$ the pressure at the origin of the coordinate system, $p_{L}$ the Lagrangian pressure, $\nabla_{L}$ the gradient operator in the Lagrangian **a** space, g the gravity, ${\mathbf{x}}_{L} \left( {{\mathbf{a}},t} \right)$ the position at the instant $t$ of a plane that moves with the fluid particles with a Lagrangian velocity ${\mathbf{v}}_{L} = \frac{{\partial {\mathbf{x}}_{L} }}{\partial t}.$

Given the space laterally unconfined, it is assumed that the Lagrangian pressure $p_{L}$ is equal to $p_{0}$ and ${\mathbf{v}}_{L} = 0$. The Langevin radiation pressure at the interface between two fluids can be calculated, considering an ultrasonic excitation normally incident to the interface from the fluid downward (numbered 1, fluid 2 being located above the interface). Using cylindrical coordinates in Eq. (1) the ARP can then be determined from the acoustic energy in both reflection and transmission. The resulting expression of the ARP at the interface is given as follows^42^:

2 $$\Pi \left( {r,t} \right) = \frac{2}{{\rho_{1} c_{1}^{2} }}\frac{{\rho_{1}^{2} c_{1}^{2} + \rho_{2}^{2} c_{2}^{2} - 2\rho_{1} \rho_{2} c_{1}^{2} }}{{\left( {\rho_{1} c_{1} + \rho_{2} c_{2} } \right)^{2} }}p_{i}^{2} \left( {r,t} \right),$$

where $\rho_{1} \;{\text{and}}\;\rho_{2}$ are the densities of respectively media 1 and 2, and $p_{i} \left( {r,t} \right)$ denotes the incident spatiotemporal pressure field at the interface. From Eq. (2) it can be observed that the force along the r-axis varies with the square of the incident pressure $p_{i} \left( {r,t} \right)$. The shape of the resulting interface deformation is expected to be proportional to the square of the pressure field distribution along the r-axis^39,42^.

### Tracking the surface displacement

Experimentally, the main challenge lies in measuring the entirety of the displacement of an optically transparent interface such as air–water. Classic experiments to observe the surface displacement induced by ARP are based on the use of a High-Speed Camera^42–44^. Camera based devices are exposed to several limitations, such as their field of view, which prevents access to the negative amplitudes of the surface displacement and to the 3D shape of the interface deformation. Also, the lighting condition and the tilt imposed to the camera reduces the accuracy of the measurements^39^. Other experimental strategies have been explored, such as the use of a laser probe^45–47^.

These methods are still restricted to study the time evolution of the interface deformation characterization on a few places. In the present work, tracking the surface displacement of a transparent interface has been possible through the use of a polychromatic confocal sensor^48,49^ (see Methods). A white light is transmitted from the controller to the sensor head through an optical fiber. The light is then focused by a chromatic dispersive lens in the displacement probe, at different focal lengths depending on the wavelength. The position of the interface is determined by the analysis of the spectrum of the light backscattered by the surface to the spectrometer in the probe controller. More specifications about the chromatic confocal displacement sensor are given in Supplementary Table 1.

**Figure 1:**
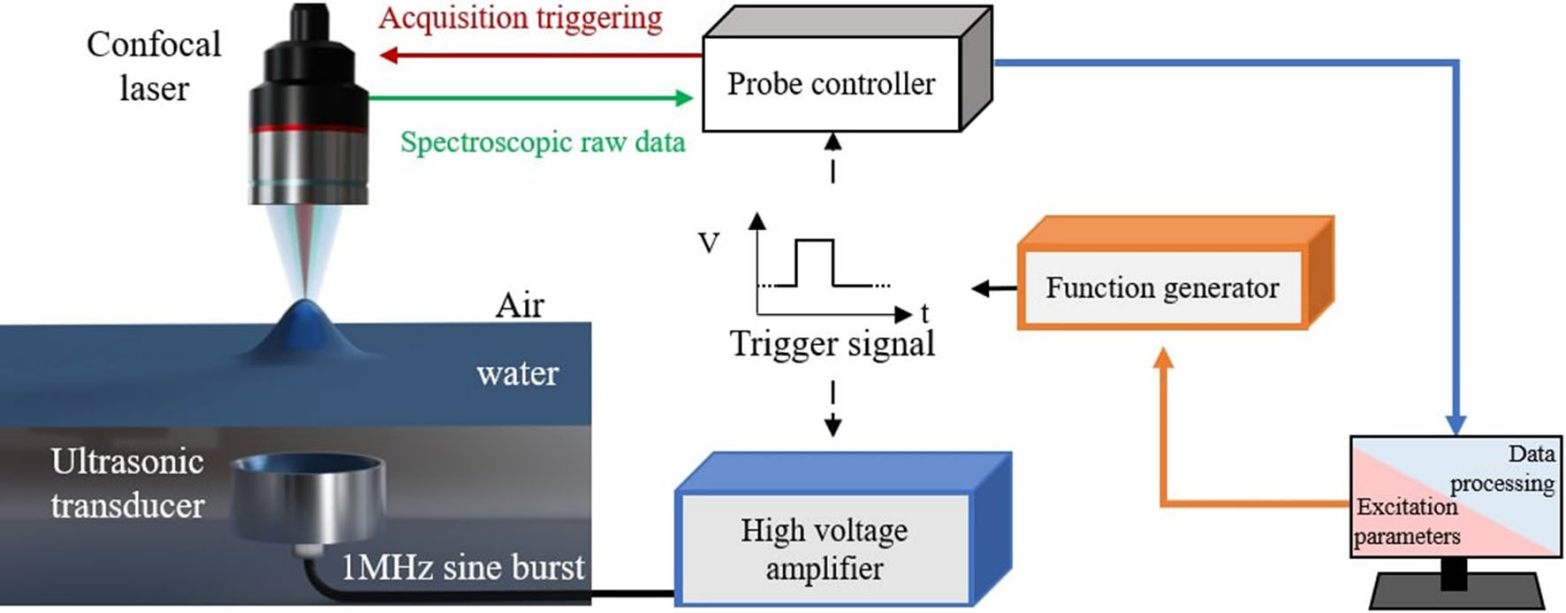
Space–time tracking of the surface elevation. A confocal displacement sensor CL-P070 is used to measure the time evolution of the water–air surface displacement induced by the ARP. The confocal laser measurement is synchronized with an ultrasonic excitation generated by an amplifier delivering a 1 MHz electrical burst to an immersed transducer. Triggering is performed through an electrical pulse from a function generator remotely driven by a computer.

The experimental setup used in this work is described in Fig. 1 and an in-depth description is given in the method section. The ultrasonic emitter is immersed in a tank filled with water at ambient temperature. Two 1 MHz piezoelectric transducers of different shapes (spherically focused and planar active area) are used to form different spatial distributions of ultrasonic beam incident to the interface. The transient acoustic excitation is a burst made of 50 sine periods at 1 MHz (total excitation duration $\tau =$ 50 μs). The input voltage is set to obtain a peak acoustic pressure $p_{i0}$ of 2.1 MPa at the water–air interface, which corresponds to the non-linear quadratic regime. A 2D scan of the surface displacement is performed using the confocal displacement sensor to track the surface motion. To verify their accuracy, experimental results are compared to simulations using FEM to solve the Navier–Stokes equation. The acoustic radiation pressure described by Eq. (2) is applied on the free surface boundary condition, as well as the surface tension ($\sigma = 72\;{\text{mN}}\;{\text{m}}^{ - 2}$ for water–air interface). The gravity is included in the model as a force term directed in the *z-* direction. The aim is to demonstrate that different pressure fields can determine dynamics of a liquid surface under the effect of ARP. Transducers were selected to generate specific deformation for different spatiotemporal shaping and applications.

### Shape of the interface deformation

**Figure 2:**
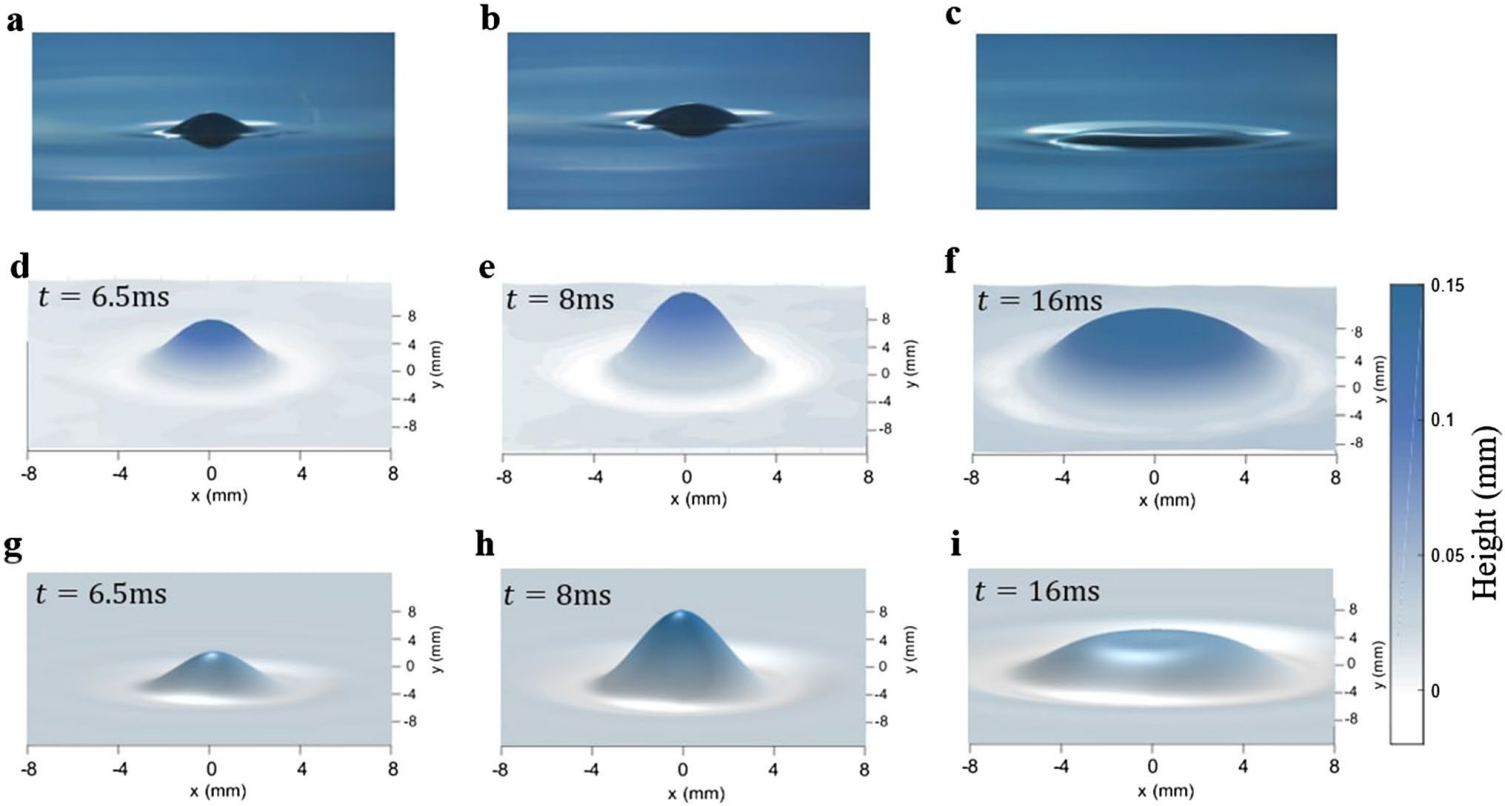
3D characterization of the interface deformation induced by a transient acoustic excitation. Three phases are highlighted: rise (**a**,**d**,**g**), maximum (**b**,**e**,**h**),and decrease (**c**,**f**,**i**). (**a**–**c**) Pictures of the deformation shot with a reflex camera for $p_{i0} = 3.5\;{\text{MPa}}$ and $\tau = 50\;{\text{ms}}$. (**d**–**f**) Experiment results of a 2D scan for $p_{i0} = 2.1\;{\text{MPa}}$ and $\tau = 50\;{\text{ms}}$. (**g**–**i**) 3D view of a simulation results for $p_{i0} = 2.1\;{\text{MPa}}$ and $\tau = 50\;{\text{ms}}$. The time evolution of the measured and simulated surface deformation are available in Supplementary Video 1a,b.

The influence of the incident transient excitation parameters on the shape of the deformation is investigated experimentally and numerically. The model allows focusing on both spatial and temporal evolution of the deformation considering the formulation of the input excitation. Three different time steps can be distinguished as shown on the pictures taken with a Nikon D5600 camera (Fig. 2a–c): rise, maximum and decrease of the deformation height. Experimentally, in the case of the focused transducer, and for $p_{i0} = 2.1\;{\text{MPa}}$ and $\tau = 50\;{\text{ms}}$, a scan of the surface is performed using the confocal displacement sensor to track the surface displacement. The perturbation of the surface results in a circular protrusion around a vertical axis whose radius increases with time during the three phases. Afterward, during the release, the perturbed surface returns to its initial level due to the transient excitation.

The 2D deformation shapes depicted in Fig. 2 presents a comparison of laser measurements (Fig. 2d–f) and FEM simulation results (Fig. 2g-i) at three different times: t = 6.5 ms (Fig. 2d,g), t = 8 ms (Fig. 2e,h) and t = 16 ms (Fig. 2f,i). A good agreement is found between the measurements performed with the confocal displacement sensor and the FEM simulation. At *t* = 8 ms, both experiments and simulations exhibit a deformation height of 0.13 mm and a deformation width of 4 mm radius for a 2.1 MPa incident pressure at r = 0. It highlights that for a water–air interface, modelling the surface displacement in space and time caused by an incident ultrasonic beam can be accurately performed using Eq. (2).

**Figure 3:**
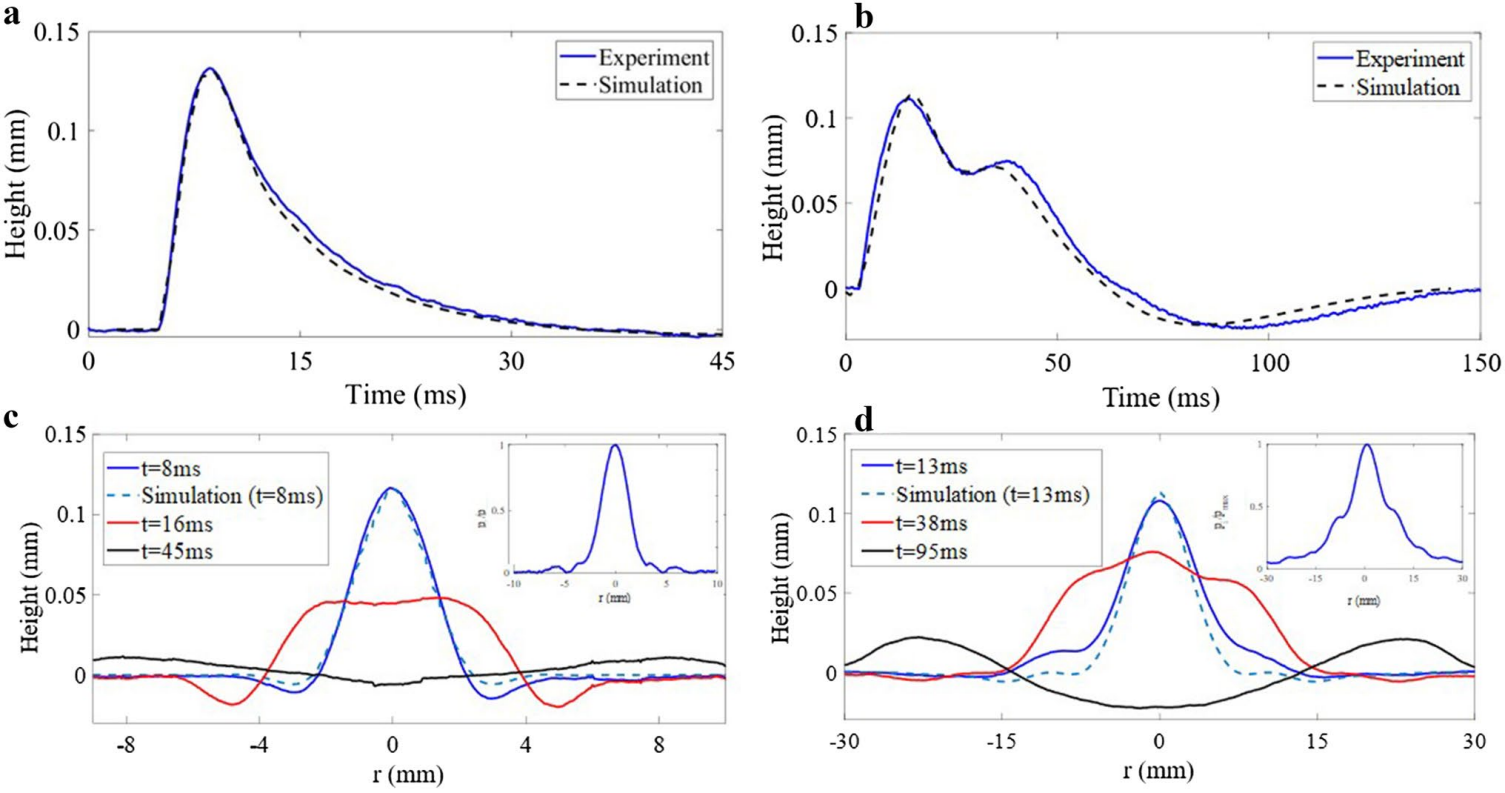
Time dependences and spatiotemporal shapes of the deformation for different input pressure fields. Experiments (solid lines) and FEM simulations (dashed lines) of the time evolution of the point located on the transducer axis are compared for (**a**) a spherical and (**b**) a planar transducer. (**c**) 2D shape of the interface deformation resulting from a spherical transducer at its maximum height (*t* = 8 ms), during the decrease (*t* = 16 ms) and for its minimum height (*t* = 45 ms). (**d**) 2D shape of the interface deformation resulting from a planar transducer at its maximum height (t = 13 ms), during the decrease (*t* = 38 ms) and at its minimum height (*t* = 95 ms). Insets in (**c**,**d**) are the linear input pressure fields from hydrophone measurements for each transducer.

The time evolution of the height of the water–air interface deformation on the transducer axis (r = 0, z) induced by the ARP is presented in Fig. 3a,b for two different transducers. The simulations (black dashed lines) are in good agreement in time and in height with experimental results. Looking at the temporal evolution for a spherical transducer (Fig. 3a, solid blue line), the water surface reaches a maximum height of 125 μm in 4.7 ms (the excitation stops after 0.05 ms), to then decrease to its initial state at 45 ms. The behaviour observed for a planar transducer show different dynamics (Fig. 3b). The surface deformation reaches its maximum height after 13 ms and then reaches a second peak value at t = 38 ms. The time of release is longer for a spherical transducer (approximately 140 ms). This difference is due to the spatial distribution of the pressure at the interface (insets Fig. 3c,d). For the planar transducer, the pressure at the interface is spread over a radius of 12 mm diameter, resulting in a smaller energy density than the spherical transducer where all the pressure is distributed in a radius of 2 mm. Also, as shown by Fig. 3d, side waves induced by the finiteness of the active area of the planar transducer, have significant effect. These side waves generated at ${\text{r}} = \pm 10\;{\text{mm}}$ propagate and then collides at r = 0, interfering with the wave induced by the main lob of the incident pressure field (the solid red line on Fig. 3d.). It results in a second rise of the interface and an extended relaxation time, as depicted by the solid blue line at 38 ms on Fig. 3b. This effect may be reduced using a more viscous fluid, as the motion of the surface would be damped, thereby preventing the interference of side waves.

For the spherical transducer, it can be observed that the shape of the deformation during the rise (at t = 8 ms*)* is the same as the pressure field distribution as highlighted in Fig. 3c (solid blue line). The evolution over time highlighted on Fig. 3c,d, shows that the width is increasing during the time of rise (2 mm width at − 6 dB for t = 8 ms*),* followed by a further widening during the decrease (6 mm at -6 dB for t = 16 ms). Finally, deformation splits into a quasi-axisymmetric circular capillary wave, centred around (r = 0, z) as denoted by the 2D representation (black solid line) on Fig. 3c,d. A good agreement can be observed at the time of maximum elevation in Fig. 3c,d between experiments (blue solid lines) and simulation (blue dashed lines).

### Time reconfigurability

**Figure 4:**
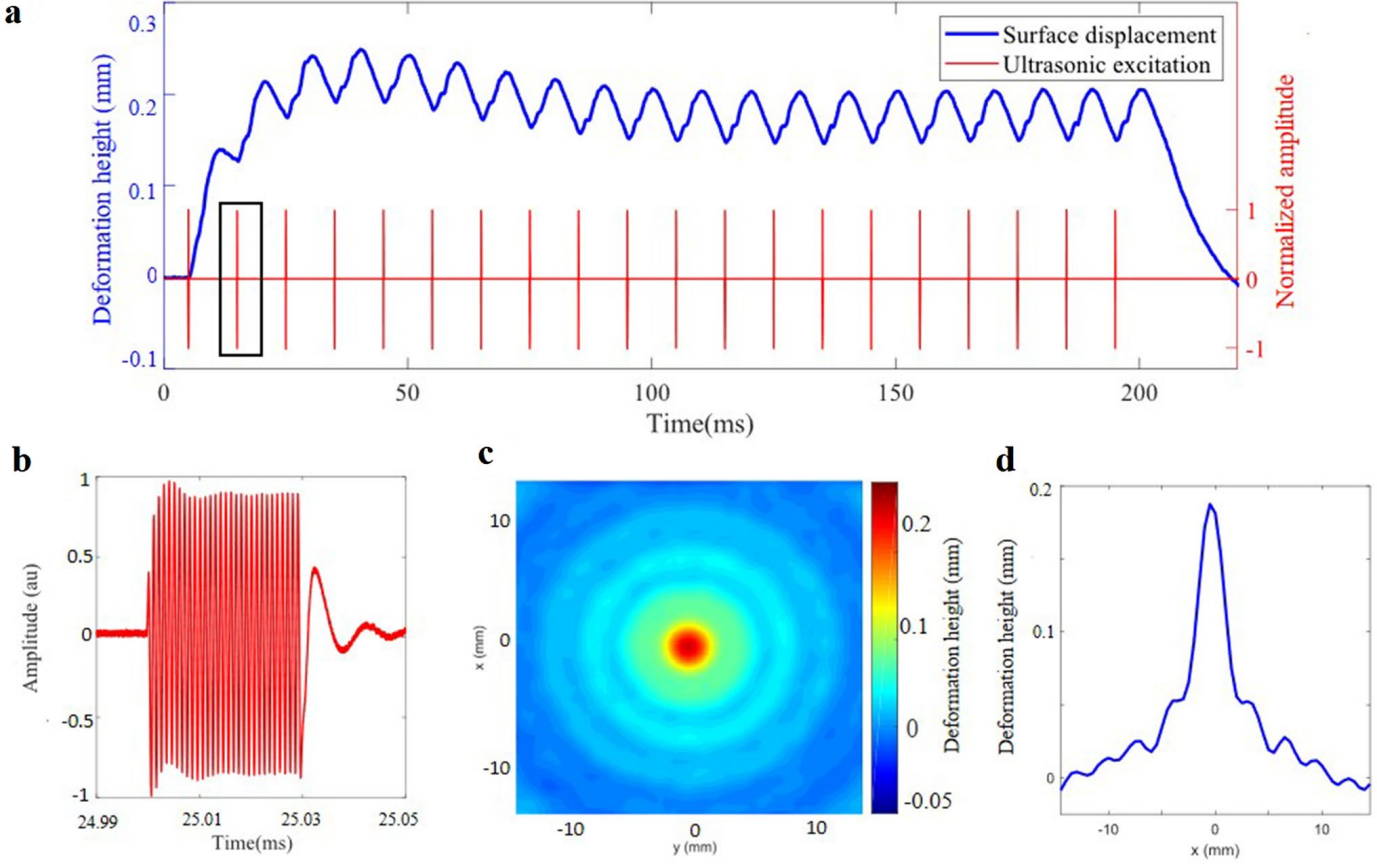
Influence of the time sequence of the excitation. (**a**) The time evolution of the surface displacement at *r* = 0 (solid blue line) induced by a periodic burst excitation (solid red line). The 1 MHz 30 period sine burst (highlighted with a black frame) is repeated at a frequency of 100 Hz during 200 ms, (**b**) shows an extended view of one burst. (**c**) 2D shape of the resulting interface deformation at $t = 58\;{\text{ms}}$ and (**d**) Cross-sectional profile of the interface deformation along $x$ at $y = 0$.

Dynamic shaping of the fluid interface deformation can be achieved by accurately manipulating the instantaneous distribution of the acoustic radiation pressure applied to the interface. This process is constrained by the fluid properties and gravity, as they are responsible of the passive reconfiguration of the deformation back to its initial state without the need for external energy input. As presented previously in Fig. 3a,b, complete reset of the interface deformation can be achieved by utilizing burst excitation with a sufficiently low repetition frequency. This demonstrates the possibility to achieve transient shaping of interface within a cycle of 40 ms. Another possibility is to use a series of ultrasonic bursts, with a pulse repetition frequency (PRF) high enough to prevent the deformation of the surface from returning to its initial state. Study is conducted using a spherical transducer to investigate the manoeuvrability of the surface shaping presented in Fig. 2. A 1 MHz burst consisting of 30 periods (Fig. 4b), emitted with a PRF of 100 Hz along 200 ms (solid red line on Fig. 4a), is used. The measurement of the surface displacement at $r = 0$ reported in Fig. 4a (solid blue line) shows a rise of the water global level during the first 40 ms, up to a maximum value of 250 μm. Then it decreases to a steady state which starts at 90 ms, where the height of the interface deformation oscillates around a mean height of 175 μm, with an amplitude of 50 μm peak to peak ($h_{max} = 200\;\upmu {\text{m}}$, $h_{min} = 150\;\upmu {\text{m}}$) and a period of 10 ms. The mean value of these oscillations could be greater increasing the PRF. This is due to the viscosity of water which controls the time required for the surface to return to the initial state. The surface displacement between two pulses is thereby limited and results in an increase of $h_{min}$ . Contrariwise, decreasing the PRF increases the amplitude of the oscillation and decreases the mean value until it reaches the same as the one of a single burst excitation. Hence, short reconfiguration time can be accomplished (10 ms for a PRF of 100 Hz), but it induces a reduction of the contrast $h_{max} / h_{min}$. This allows to have a fast and on-demand modulation of the surface deformation height, oscillating between two states depending on the PRF. It should be noticed that the viscosity of the fluids influences the envelope of the global surface displacement, changing the time of rise and decrease, and thus the amplitude of the oscillations around the mean height at the steady state.

The application of a periodic burst excitation induces periodic ripples on the water surface, as highlighted in Fig. 4c by the 2D top view of the surface deformations resulting from a PRF of 100 Hz at t = 59 ms. The main lobe in the center is surrounded by circular wavelets propagating outward (see Supplementary Video 2). The cross-sectional 1D signal extracted at *r* = 0 (Fig. 4d) shows that the induced ripples have a period of 10 ms and characteristic wavelength of 3 mm, which correspond to a celerity of 0.3 m/s^50^. It can be observed that the repetition of pulses induces a significant uplift at $r = 0$, which results in a depression (negative level) for $10\;{\text{mm}} < r < 15\;{\text{mm}}$. Changing the PRF enables to change the period of these ripple patterns. It is worth noting that the temporal periods induced by the PRF at *r* = 0 remain constant throughout waves propagation as depicted by Fig. 4c,d.

## Discussions

In this study, we demonstrate that a fluid interface can be remotely shaped in space and time using an ultrasonic beam, depending on the characteristics of the transient input electrical excitation. The transfer of momentum from propagating ultrasonic waves in the medium results in a force on the interface which makes water raise. We show that this surface displacement could be predicted using FEM, and accurately measured in time and space using a confocal displacement optical sensor. The good agreement between both numerical and experimental methods states that the shape of the interface deformation induced by ARP can be obtained using the spatiotemporal distribution of the input pressure field at the interface (Eq. 2).

We further compare the influence of the spatial and temporal distributions of the incident pressure field on the interface on the spatiotemporal shape of the surface using planar and spherical transducers. Based on the proposed experiment and numerical model, the time to return to the initial state is shorter in the case of the spherical transducer due to the greater energy density on the surface and to the lack of side lobes. For both transducers, the results strongly rely on the distribution of the incident pressure field exerted on the interface, exhibiting a more spread-out deformation in the case of a planar transducer.

The experimental and numerical works highlight three distinct time states of the interface deformation: rise, maximum and decrease. We show that the perturbation of the surface exhibits a circular shape whose radius increases with time during the three phases. During the release due to the transient excitation, the surface splits into a circular capillary wave and returns to its initial level due to the transient excitation.

The agility of the interface shaping relies on the time profile of the excitation. Using a spherical transducer, a full reset occurs when the pulse repetition period is greater than the time required for the surface to returns to its initial state. The viscosity, density, and surface tension of the water–air interface limit its displacement over time and so between two successive pulses. Therefore, increasing the PRF results in a shortest reconfiguration time around the average value of water lifted vertically at the transducers main axis, and reduces the $h_{max} / h_{min}$ ratio. The experimental work shows that using ultrasonic pulses with relevant repetition rate can be an effective way to pattern a fluid surface as time dependent, which can be used to study liquid surface properties^51,52^.

Our study demonstrates the potential of using ultrasonic beams to shape fluid interfaces in space and time. This work indicates that the spatiotemporal shaping of the water–air interface strongly depends on the shape of the incident pressure field emitted by the transducer. We have successfully achieved a time-dependent shaping of a water–air interface by changing the time sequence of the ultrasonic excitation.

This ability to remotely manipulate the spatiotemporal characteristics of fluid interface gives opportunities for experimental studies of time-varying media. This paves the way for a wide range of potential applications from material science to microfluidics, to the development of novel techniques for wavefront shaping and non-linear reflection in the fields of optics and acoustics.

## Methods

### Experiment

Experiments are carried out in a tank filled with water at 20 °C. Two 1 MHz piezoelectric transducer (NDT SYSTEM IDHG018) of diameter 40 mm are used successively: a spherical one focusing at 40 mm, and a planar one with a Rayleigh distance at 120 mm. More specifications on the transducers are given in Supplementary Table 2. To induce a transient acoustic excitation, an electric burst signal made of 50 sine periods of 1 µs, delivered to the transducers by an ultrasonic pulser (RITEC RAM 5000) and connected to a computer. A polychromatic confocal displacement probe CL-P070 (Keyence) with a spot diameter of 50 μm operating at a sample rate of 10 kHz is used. The horizontal spatial resolution of the scan is set at 50 μm over 401 measurement points for the spherical transducer, and to 0.2 mm over 301 measurement points for the planar one. The sensor head is placed 70 mm above the water surface, vertically in relation to the measurement spot. It allows a measurement range of ± 10 mm of the surface displacement with a vertical resolution of 0.25 µm. Data are acquired with a probe controller CL-3000 (Keyence). The pulser and the probe controller are triggered by an arbitrary waveform generator (Agilent 33220a) drive by a computer through MATLAB software. It is used to perform a single period if square wave excitation, using a 1 Hz pulse repetition frequency (PRF), or with a 100 Hz PRF to investigate shortest reconfiguration times of the interface.

### Simulation

The Finite Element Analysis is performed using the Comsol Multiphysics software. The module “Laminar Two-Phase Flow, Moving Mesh” is used to solve the Navier–Stokes equation for incompressible flow. This method enables to study the time evolution of the displacement of the interface between two fluids. The model consists in a 2D geometry and is bounded by two types of boundaries. The top boundary is modelled with a free surface condition, including the surface tension between the two fluid σ and two no-slip wall boundaries specifies the stationary solid walls on the bottom and right sides of the fluid domain. The geometry of the sample is large enough so the surface waves resulting from the interface deformation do not interfere with the walls during the simulation time (150 µs), with the left and right-sided wall located at ± 50 mm from the centre of the domain, and the bottom at a depth of 5 mm. The gravity is considered as a force directed downward (with $\vec{g} = - \rho g\vec{z}$, $g = 9.81\;{\text{m}}/{\text{s}}^{2}$). The ARP denoted by Eq. (2) is modelled as an external pressure at the free surface boundary condition and is evaluated from the incident pressure field spatial distribution $p_{i} \left( {r,t} \right)$ which is set using the experimental results from hydrophone characterisation of the pressure field at the focal point of both spherical and planar transducers (Fig. 3c,d).

### Supplementary Information

Supplementary Tables.

Supplementary Video 1a.

Supplementary Video 1b.

Supplementary Video 2.

## Data Availability

The datasets used and/or analysed during the current study available from the corresponding author on reasonable request.
