# Supplementary material for: Contactless deformation of fluid interfaces by acoustic radiation pressure
Source: Sci Rep. 2023 Sep 7;13:14703. doi: 10.1038/s41598-023-39464-0 (PMC10485005; doi:10.1038/s41598-023-39464-0)
Supplement: Supplementary file 1 — Supplementary Tables. [file 41598_2023_39464_MOESM1_ESM.docx]

Supplementary information

**Remote shaping of fluid interfaces by acoustic radiation pressure**

Félix Sisombat^1^, Thibaut Devaux^1^, Lionel Haumesser^1^, Samuel Callé^1^

^1^*GREMAN UMR 7347, Université de Tours, CNRS, INSA CVL, 41000 Blois, France*

**Supplementary Table 1:** Confocal displacement sensor CL-P070 specifications

| **Parameters** | **Value** | **unit** |
| --- | --- | --- |
| Spot diameter | ø50 | μm |
| Dynamic | ±10 | mm |
| Resolution | 0.25 | μm |
| Sampling period | 100 | µs |
| Distance from the interface | 70 | mm |

**Supplementary Table 2:** Transducers specifications

|  | Spherical |  |  |  | Planar |  |
| --- | --- | --- | --- | --- | --- | --- |
| **Parameters** | **Value** | **unit** |  | **Parameters** | **Value** | **unit** |
| Frequency | 1 | MHz |  | Frequency | 1 | MHz |
| Focal length | 40 | mm |  | Focal length | 120 | mm |
| Diameter | ø40 | mm |  | Diameter | ø40 | mm |
| F - number | 1 |  |  | F - number | 3 |  |

**Supplementary Video 1a:** Experimental 2D scan results performed with the confocal displacement probe, of the interface deformation induced by a transient acoustic excitation, with an incident pressure $p_{i0}= 2.1MPa$ and an excitation duration $\tau=50ms.$

**Supplementary Video 1b:** Simulation results of the interface deformation induced by a transient acoustic excitation, with an incident pressure $p_{i0}= 2.1MPa$ and an excitation duration $\tau=50ms.$

**Supplementary Video 2:** Experimental 2D scan results performed with the confocal displacement probe, of the interface deformation induced by a periodic burst acoustic excitation. The 1MHz 30 period sine burst is repeated at a frequency of 100Hz during 200ms, with an incident pressure $p_{i0}= 2.1MPa$ and an excitation duration $\tau=50ms.$
